# Local decision makers’ awareness of the social determinants of health in Turkey: a cross-sectional study

**DOI:** 10.1186/1471-2458-12-437

**Published:** 2012-06-15

**Authors:** Evci (Kiraz) Emine Didem, Ergin Filiz, Okur Orhan, Saruhan Gulnur, Beser Erdal

**Affiliations:** 1 Department of Public Health, Adnan Menderes University, School of Medicine, Aydin, Turkey

**Keywords:** Social determinants of health, Healthy cities, Questionnaire, Local awareness, Community-based studies

## Abstract

**Background:**

Social determinants have been described as having a greater influence than other determinants of health status. The major social determinants of health and the necessary policy objectives have been defined; it is now necessary to evaluate the effectiveness of these policies. Previous studies have shown that descriptions of the awareness level of citizens and local decision makers, practice-based research and evidence, and intersectoral studies are the best options for investigating the social determinants of health at the community level. The objective of the present study was to define local decision makers’ awareness of the social determinants of health in the Aydin province of Turkey.

**Methods:**

A total of 53 mayors serve the Aydin city center, districts and towns. Aydin city center has 22 neighborhoods and 22 headmen responsible for them. The present study targeted all mayors and headmen in Aydin - a total of 75 possible participants. A questionnaire was used to collect the data. The questionnaire was faxed to the mayors and administered face-to-face with the headmen.

**Results:**

Headmen identified the three most important determinants of public health as environmental issues, addictions (smoking, alcohol) and malnutrition. According to the mayors, the major determinant of public health is stress, followed by malnutrition, environmental issues, an inactive lifestyle, and the social and economic conditions of the country. Both groups expressed that the Turkish Ministry of Health, municipalities and universities are the institutions responsible for developing health policy. Headmen were found to be unaware and mayors were aware of the social determinants of health as classified by the World Health Organisation. Both groups were classified as unaware with regard to their awareness of the Marmot Review policy objectives.

**Conclusions:**

Studies such as the present study provide important additional information on the social determinants of health, and help to increase the awareness levels of both local decision-makers and the community. Such studies must be considered a vital first step in future public health research on health determinants and their impact on national and international policies.

## Background

The field of the social determinants of health is concerned with key aspects of people’s lifestyles and their living and working circumstances [1]. It is concerned with the health implications of social and economic policy and the benefits that investing in health policy can bring [1].

The determinants of health are multiple and varied and include: living and working conditions; access to good quality services including health care, schools and education; environmental factors including water and air quality, environmental pollution caused by hazardous substances and emissions, urbanization, climate change, rising temperatures and sea levels, natural disasters and extreme weather conditions; behavioral factors including smoking, alcohol consumption, diet, exercise and substance abuse; and the capacity and efficiency of health systems. All of these factors have a significant impact on health [2-4].

The widely cited Dahlgren and Whitehead rainbow model of the main determinants of health is used as a framework to identify the range of social determinants [5,6]. According to this model, the determinants of health can be classified under individual lifestyle factors, social and community networks, and general socioeconomic, cultural and environment conditions. The second edition of *Social Determinants of Health: The Solid Facts*, published by the World Health Organization (WHO) [6], summarizes the social determinants of health under ten headings: (1) social gradient, (2) stress, (3) early life, (4) social exclusion, (5) work, (6) unemployment, (7) social support, (8) addiction, (9) food, and (10) transport [6].

Social determinants have been described as having a greater influence than other determinants on health status [2-4]. It is, therefore, vital that health policy tackles these determinants. In 2005 the World Health Organization established the Commission on Social Determinants of Health (CSDH) to catalyze action to tackle the unequal distribution of health within and between countries, an unequal distribution that, it argues, is the consequence of the unequal distribution of the social determinants of health [7]. In 2009 a resolution was passed at the World Health Assembly that called on the WHO and all Member States to take action on the social determinants of health. The WHO Regional Office for Europe also commissioned a regional review of the health divide and inequalities in health from July 2010 to 2012 to inform the new health policy for the Region [8].

The seminal Marmot Review into health inequalities in England was published on 11 February 2010. The report proposed a new way to reduce health inequalities in England post-2010. Central to the Review is the recognition that disadvantage starts before birth and accumulates throughout life. This is reflected in the six policy objectives and to the highest priority being given to the first objective: (1) give every child the best start in life; (2) enable all children, young people and adults to maximize their capabilities and have control over their lives; (3) create fair employment and jobs; (4) ensure a healthy standard of living for all; (5) build healthy and sustainable communities; and (6) strengthen the role and impact of ill-health prevention [9].

In October 2011, WHO and the government of Brazil organized the World Conference on Social Determinants of Health in Rio de Janeiro. The conference focused on five issues seen to be essential for effective action on the social determinants of health: (1) governance, (2) participation, (3) the changing role of the health sector, (4) the need for global action, and (5) how to monitor progress [10].

The major social determinants of health and the necessary policy objectives have been defined; it is now necessary to evaluate the effectiveness of these policies. Previous studies have shown that descriptions of the awareness level of citizens and local decision makers, practice-based research and evidence, and intersectoral studies are the best options for investigating the social determinants of health at the community level [3,11].

Heads of neighborhoods, district governors, mayors, governors and members of the provincial assembly have the responsibility of local decision making in Turkey. As heads of the smallest unit of local government, heads of neighborhoods have a close understanding of the characteristics of their neighborhoods and the people living there. Mayors are elected governors of cities that become a union through a merger of neighborhoods. The objective of the present study was to define local decision makers’ awareness of the social determinants of health.

## Methods

### Study design and participants

This cross-sectional study was conducted between September and October 2011 in Aydin, a city in western Turkey with a population of 989,862 people. A total of 53 mayors serve the Aydin city center, districts and towns. Aydin’s city center has 22 neighborhoods and 22 headmen responsible for them. The present study targeted all mayors and headmen in Aydin - a total of 75 possible participants.

The necessary conditions for becoming a headman or mayor are laid down in the *Act for Selection of Local Governments, Heads of Neighborhoods and Board of Alderman (No. 2972 of 1984)*. According to this legislation, any Turkish citizen over the age of 25 who has no obstacle to being selected according to the provisions in the act, and other acts referred to in the act, can be selected as a headman, provided they are a resident in that neighborhood or village for at least 6 months. Graduating from primary school is not a necessary condition and being literate is sufficient. According to the same Act, “Any citizen having eligibility to be selected in line with written conditions in the Constitution and Laws can be a candidate in election for the mayoralty from the list of a political party or independently” [12].

### Procedure

In general, face-to-face interviews are the preferred data collection method for studies conducted in Turkey. This method is, however, more expensive compared with other methods (such as observation or postal, fax, and e-mail surveys), more time consuming, more difficult to conduct, and the most open to interviewer bias. Despite these drawbacks, this method is preferred because of the instant response, and the opportunity to clarify questions and probe responses, which increases the likelihood of obtaining accurate and rich data [9]. In the present study, a questionnaire was used to collect data and was faxed to the mayors and administered face-to-face with the headmen of neighborhoods.

The questionnaire consisted of 14 questions and covered the following topics: (1) sociodemographics (nine questions); (2) general opinions on health determinants (one open-ended question); (3) policy making institutions on health (one open-ended question); (4) social determinants of health and their impact (two close-ended questions); and (5) recommendations for intervention against negative impacts.

A list, including contact information for the mayors and headmen, was obtained from the Aydin Governorship, Directorate of Local Government. As some of the municipalities are between 35 to 120 km away from Aydin's city center, the researcher decided to fax the questionnaire to the mayors to save time, manpower and money. Contact was initially made with the secretary of each mayor to set up a telephone appointment with the mayor. Each mayor was then called at the date and time given by the secretary. During the telephonic conversation with each mayor, they were provided with the necessary information pertaining to the study and verbal informed consent for participation was obtained. The questionnaire was faxed to each mayor on the same day that consent was obtained. Each mayor was given a date by which to return the questionnaire. If the questionnaire was not returned by the deadline, or an incomplete questionnaire was returned, the questionnaire was faxed again. Fifty mayors returned a questionnaire via fax. However, eight of the questionnaires were excluded from the analysis as only the first eight questions had been answered.

Heads of neighborhoods were visited at one of the official buildings in their neighborhood by one female and one male researcher trained to administer the questionnaire. Information pertaining to the study was read to potential participants and verbal informed consent was obtained. An appointment was then made to administer the questionnaire at a time convenient to the headman. The neighborhoods were either within walking distance of each other or a maximum of a half an hour away by public transport. Hence, data were collected from headmen through face-to-face interviews. All 22 headmen were interviewed.

### Ethical considerations

The study protocol was designed in compliance with the Helsinki declaration (Seul, October 2008) and approved by Adnan Menderes University Rectorate connected with approval of the Provincial Local Administration Committee of Aydin Governorship (ID for study with headmen of neighborhoods 23.09.2011/6832; ID for study with mayors 04.10.2011/7055) and consent was obtained from all participants. The department of public health of medical faculty of Adnan Menderes University is responsible for the design and conduct of the study.

### Statistical analysis

SPSS 19.0 for Windows® software (IBM, Serial Number 10241440) was used for statistical analysis of the data. Descriptive statistics were used to summarize the data set. Thereafter, two awareness scores were calculated. The first awareness score represents awareness of the World Health Organization’s ten social determinants of health. This awareness score was calculated for the mayors as a group and the headmen as a group, rather than for individual participants. If, for each of the ten determinants, 90% or more of the respective group was aware of that determinant, then a score of one was awarded. If less than 90% of the group was aware of the determinant, then a score of zero was awarded. The scores for each of the ten determinants were then summed to provide a total awareness score for each group. If the total score was between zero and five, then the group was considered to be unaware; if the total score was between six and ten, then the group was considered to be aware. The policy objectives awareness score was also calculated for each of the two groups as a whole, rather than for individuals. Each group made recommendations for addressing the social determinants of health. These recommendations were then compared with the six policy objectives of the Marmot Review to determine if there was any overlap. A score of one was awarded for each of the Marmot Review policy objectives that were reflected in the respective groups’ recommendations. These scores were then added to obtain a total score for awareness of the policy objectives. If the total score was less than six, then the group was considered to be unaware. If the total score was six, then the group was considered to be aware (Table 1).

**Table 1:** Comparison of participants’ recommendations with Marmot Review policy objectives

**Marmot Review**		
***Policy Objectives***		
	**Mayor (n = 42)**	HN* (n = 22)
Give every child the best start in life	1	0
Enable all children, young people and adults to maximize their capabilities and have control over their lives	0	1
Create fair employment and jobs	1	0
Ensure healthy standard of living for all	1	1
Build healthy and sustainable communities	1	1
Strengthen the role and impact of ill health prevention	1	1
Awareness score**	**5**	**4**

* Head of neighborhood (HN).

** Awareness score (AS): For every policy objective of the Marmot Review that is present among the participants’ recommendations, then score is 1;total score ‹6 = unaware, total score 6 = aware.

## Results

### Sociodemographics

The mean term of office for mayors was 5.26 ±3.93 years (range 6 months-22 years) and for heads of neighborhoods was 8.39 ±7.67 years (range 1–27 years). The mean age of mayors was 50.55 ±6.71 years (range 33–67 years) and of heads of neighborhoods was 57.50 ±8.14 years (range 42–73 years). Of the 64 participants, only two mayors and one head of neighborhood were female. All participants were married. Some of the sociodemographic characteristics of the study population are provided in Table 2.

**Table 2:** Sociodemographic characteristics of the study population

	**Mayors (n = 42)**	**Heads of neighborhood (n = 22)**
**Gender**		
Female	2	1
Male	40	21
**Age Group**		
‹50	16	4
50-60	21	8
›60	5	10
**Educational status**		
Primary School	8	8
Secondary and high school	13	9
University and above	21	5
**Term of office**		
‹5 years	23	11
≥5 years	19	11
**Before current position, any official responsibility**		
Yes	20	22
No	22	0
**Received training on project cycle**		
Yes	8	2
No	34	20
**Read newspaper daily**		
Every day	37	17
Rare	5	5
**Use internet daily**		
Every day	29	10
Rare	13	12
**Follow the** ***Official Gazette*** *		
Every day	11	0
Rare	24	1
Never	7	21

* The official daily newspaper where all new governmental regulations and announcements are published.

### Determinants of health

A total of five questions were asked in this section. The responses to each of the five questions are presented below.

(1) **What affects the health of society?**Headmen identified the three most important determinants of public health as environmental issues (n = 8), addictions such as smoking and alcohol (n = 8) and malnutrition (n = 7). According to the mayors, the major determinant of public health is stress (n = 20), followed by malnutrition (n = 18), environmental issues (n = 16), an inactive lifestyle (n = 16), and the social and economic conditions of the country (n = 16).

(2) ***Which institutions are responsible for developing health policy?***Both headmen and mayors expressed that the Turkish Ministry of Health (n = 21 and n = 34 respectively), municipalities (n = 5 and n = 11 respectively) and universities (n = 2 and n = 9 respectively) are responsible for developing health policy.

(3) ***Which of the following has an effect on health?***Both headmen and mayors were presented with the WHO classification of the social determinants of health [6]. Participants were asked to indicate which of the ten determinants they believe has an effect on health. Responses from headmen and mayors, as well as their awareness score values are shown in Table 3. Headmen were found to be unaware (awareness score = 5) and mayors were aware (awareness score = 8) of the social determinants of health (Table 3).

(4) ***Mark those items you believe are important aspects of each determinant***Participants were provided with a number of sub-items for each of the 10 WHO determinants and were asked to indicate which of the sub-items are important aspects of that determinant. Multiple responses were allowed. The sub-items provided were derived from the second edition of *Social Determinants of Health: The Solid Facts*[6]. Distribution of the three most indicated options for the headmen and mayors for each determinant is provided in Table 4. The majority of the headmen selected “feeling out of control about work” (86.4%) as an important aspect of the determinant work. Headmen indicated “decline in social support = decline in prosperity” (72.7%) as an important aspect of social support, and “poor economic circumstances” (59.1%) as an important aspect of social gradient. Mayors appeared to find it difficult to identify the important sub-items as they assigned equal significance to all the given options. Only “stress” had a value under 50%.

(5) ***What are your recommendations for addressing the determinants of health? What should be done?***Headmen and mayors expressed their opinions in an open-ended question regarding measures to address the determinants of health. The following recommendations were made: increase the level of education (17%); make attempts to protect the environment (16%); protect health and develop preventive medicine (14%); improve nutritional habits (12%); increase physical activity (10%); decrease stress (7%); prevent addictions such as smoking and alcohol (6%); provide socioeconomic development (6.0%); disseminate public transport in the city (3); decrease unemployment (3.0%); improve city health (3%); and provide social justice (3%). The recommendations of the headmen and mayors were compared with the six policy objectives of the Marmot Review to determine if there was any overlap. Headmen made recommendations consistent with four of the six objectives, but made no recommendations consistent with giving every child the best start in life or creating fair employment and jobs. Mayors made recommendations consistent with five of the policy objectives, but did not make recommendations related to enabling all children, young people and adults to maximize their capabilities and have control over their lives. Both groups were classified as unaware according to their awareness score, as both scored less than six.

**Table 3:** Factors reported as having an impact on health

	**Mayors (n = 42)**		**Heads of neighborhood (n = 22)**	
**Social gradient**	**n**	**%**	**n**	**%**
Yes	42	100.0	20	90.9
No	0	0	2	9.1
Awareness score*	*1*		*1*	
**Stress**				
Yes	40	100.0	22	100.0
No	0	0	0	0
*AS*	*1*		*1*	
**Early Life**				
Yes	40	97.6	17	77.3
No	1	2.4	5	22.7
*Awareness score*	*1*		*0*	
**Social Exclusion**				
Yes	38	95.0	19	86.4
No	2	5.0	3	13.6
*Awareness score*	*1*		*0*	
**Work**				
Yes	40	97.6	19	86.4
No	1	2.4	3	13.6
*Awareness score*	*1*	*0*		
**Unemployment**				
Yes	41	100.0	22	100.0
No	0	0	0	0
*Awareness score*	*1*		*1*	
**Social support**				
Yes	33	84.6	17	77.3
No	6	15.4	5	22.7
*Awareness score*	*0*		*0*	
**Addiction**				
Yes	42	100.0	21	95.5
No	0	0	1	4.5
*Awareness score*	*1*		*1*	
**Food**				
Yes	40	95.2	22	100.0
No	2	4.8	-	-
*Awareness score*	*1*		*1*	
**Transport**				
Yes	37	88.1	16	72.7
No	5	11.9	6	27.3
*Awareness score*	*0*		*0*	
**Total awareness score**	**8**		**5**	

Awareness score: If percentage of yes for the determinant ‹90%, then score is 0; if ≥90%, score is 1. Total score: 0–5 = unaware, 6–10 = aware.

**Table 4:** Sub-items reported as important aspects of the ten WHO determinants

**WHO** ***Social determinants of health***	HN* (n = 22)
**Social gradient**	
Poor economic circumstances	13
Stressful life	5
Psychological wear	1
Changing jobs and facing redundancy	1
**Stress**	
Social isolation	1
Increased cardiovascular disease risk	1
Increased cerebrovascular disease risk	1
Immune system weakness	1
Increased depression risk	1
**Early Life**	
Inadequate income of family	11
Low educational attainment of family on bring up children	5
Maternal malnutrition during pregnancy	3
**Social Exclusion**	
Poverty	7
Facing racism and discrimination	2
Exclusion of psychiatric cases or mentally handicapped	1
**Work**	
Out of control over her/his own work	19
Stress at work	9
Inadequate value of labor	3
**Unemployment**	
Financial problems, debt expansion	11
Increased depression and worry	6
Chronic stressors	2
**Social support**	
Decline in social support = decline in prosperity	16
Lower social cohesion	9
Experience more depression	6
**Addiction**	
Violence	9
Social deprivation	4
Increase in accidents	3
Financial problems	3
**Food**	
Cancer	7
Overconsumption = obesity	4
Diabetes	1
Degenerative eye disease	1
Consumption of cheaper processed foods by the poor	1
**Transport**	
To provide exercise and reduce obesity, diabetes and cardiovascular disease	7
Exercise decreases risk of depression	7
Public transport stimulates social interaction	6
Public transport reduces air pollution	6
Cycling and walking provide exercise	6
Cycling and walking make little noise	2
**WHO** ***Social determinants of health***	**Mayors (n = 42)**
**Social gradient**	
Stressful life	33
Psychological wear	23
Poor education	22
**Stress**	
Increased depression risk	23
Lack of control over home	22
Lack of control over work	17
Immune system weakness	17
**Early Life**	
Inadequate income of family	29
Maternal smoking	20
Low educational attainment of family	20
Low educational attainment of family on bring up children	18
**Social Exclusion**	
Exclusion from employment possibilities	32
Poverty	22
Exclusion of psychiatric cases or mentally handicapped	17
**Work**	
Stress at work	30
Inadequate value of labor	19
Lack of social support	17
**Unemployment**	
Financial problems, debt expansion	34
Increased depression and worry	28
Feel insecurity	24
**Social support**	
Decline in social support = decline in prosperity	31
Experience more depression	29
Lower social cohesion	27
**Addiction**	
Violence	35
Social deprivation	30
Increase in accidents	28
Suicide	28
**Food**	
Overconsumption = obesity	34
Consumption of cheaper processed foods by the poor	25
Increased cardiovascular disease	24
**Transport**	
Public transport reduces accidents	34
Cycling and walking provide exercise	30
Cycling and walking make little noise	27

*Heads of neighborhood.

*Multiple responses were given.

## Discussion

The present study aimed to explore local decision makers’ awareness of social health determinants and their impact on health at a local level. In the current study, there were significantly fewer female decision makers in both groups, and headmen were older than mayors. More mayors than headmen indicated an education level of university and above. Some decision makers had been in office for more than one term (19 mayors, 11 headmen). Just over 50% of mayors had no previous official responsibility and both groups lacked training in project management. Reading a newspaper daily was more common than daily Internet use for both groups. Headmen reported not following the *Official Gazette*; which contains all new governmental regulations and announcements.

The results from a headmen survey conducted in the Izmir province, which is in the same region as Aydin, found that 88.6% of headmen were male and 11.4% were female; 84.0% were 46 years old or older; and most were high school graduates (55.7%). According to the headmen of Izmir, who have a similar demographic profile to the headmen of Aydin, the most important problems in their neighborhoods were zoning issues (11.4%); lack of infrastructure and transportation (6.4%); and lack of schools, parking and green areas (4.3%). Of the Izmir headmen, 23.0% had not attempted to improve these problem areas. When the top ten recommendations for improvement made by the Izmir headmen were examined, the most commonly mentioned were the need for more parking, the construction of health centers and road construction [13].

As of 2009, there are 213 municipalities in Turkey and according to the poll results; the number of female mayors who took office has increased from 18 following the 2004 local elections to 26 following the 2009 local elections. A study conducted in 2005 among the mayors of all the smaller cities in Turkey reported a similar sociodemographic composition as the sample in the present study. According to that study, 38.4% of the 742 mayors were 46–55 years old, 36.1% were 36–45 years, and 11.5% were 56+ years old. Of the mayors who participated in that study, 66.9% were primary-middle-high school graduates, 31.7% had a university qualification, and 1.1% had a postgraduate degree. Of the mayors, 37.6% had been in office for two or more terms [14]. In that study, whilst mayors saw the need for change, this did not translate into action [14].

Turkey has been part of the World Health Organization Healthy Cities Project since 1993. As part of this project, awareness raising and training on basic and advanced subjects has been offered since 2000 for mayors who are members of the national network. However, the national network only has 42 members and training on the social determinants of health is, therefore, only reaching a limited number of mayors. Four municipalities included in the present study are members of this network. The social determinants of health were the main topic on the agenda for the national network meeting held from 22 to 24 September 2011.

Muntaner and Chung have discussed the difficulties of conducting epidemiological studies on the social determinants of health [15]. We had great difficulty finding studies with which to compare the results of the present study. No similar studies investigating the views of decision makers in Turkey on health and the determinants of health were found. Comparing the results of the present study to studies conducted in other countries is complicated by the fact that the mechanisms of local government are very different. Comparison is also difficult as the majority of studies on the social determinants of health have been conducted on medical subjects [16,17]. For example, studies have been conducted on coronary disease and cancer, polio, tuberculosis, HIV, Hepatitis, STDs, children’s health and physical activity [18-25].

The Rio Political Declaration on the Social Determinants of Health, which emanated from the World Conference on Social Determinants of Health held in Brazil in October 2011, firmly placed social determinants of health on the agenda of the health sector, but also on the agendas of all the stakeholders and scientists [26,27]. The present study was conducted in 42 of the 53 municipalities in Aydın and all headmen in the city center participated. The views of the headmen and mayors who participated can, therefore, be regarded as representative of the whole province. Both groups felt that malnutrition was a determinant of health and the Ministry of Health was seen as primarily responsible for the development of health policy. Headmen were not sufficiently aware of the social determinants as summarized by the WHO, and their recommendations also revealed this lack of awareness. Whereas mayors were aware of the social determinants of health, their recommendations did not speak to that awareness. Their high awareness may be attributed to their attendance at the various seminars, conferences or training sessions on this issue held as part of the healthy cities project. Nevertheless, this awareness is not sufficient impetus for them to develop policy objectives and recommendations.

## Conclusion

As a result of Aydin study, there is a still a lack of awareness of the social determinants of health, and an even greater lack of awareness of the policy objectives regarding the social determinants of health. What should be the implications?

Training? Is training sufficient to ensure action?

Suggesting the use of software tools for decision making?

The use of local level data and analysis thereof?

Local decision makers need to have the ability to compare sources of data?

Local decision makers need to conduct impact assessments?

Clearly, a visionary approach is needed for attempts to solve local problems. For decision makers, vision developing tools are accessible statistical data, reporting from these data and resultant recommendation packages. Also, for decision makers, the correct decision-making tool is the capacity of comparison and impact assessment. Of course, use of the capacity will require an adequate level of education.

Health has become a high level priority on the agenda of local governors, along with concepts such as urban health and healthy cities. These concepts are being researched and evidence is being collected through academic and community-based studies. This evidence will guide the social interventions of decision makers and will act as a monitoring and evaluation tool. Studies such as the present one provide important additional information on the social determinants of health and help increase the awareness levels of local decision makers and the community. Such studies are a vital first step in future public health research on health determinants and their impact on national and international policies. Future studies should be conducted in collaboration with local health practitioners, local professionals on related issues, policy-makers and citizens. Raising awareness of the importance of the social determinants of health among policy makers is important but, in itself, not sufficient unless it translates into practice [28]. Training and education on the social determinants of health should be extended to other practitioners, policy makers, stakeholders and professionals, such as urban planners, transport planners, teachers and architects. To aid this dissemination, there is an urgent need to develop a common language of the social determinants of health, and training and learning resources focused thereon.

## Competing interests

The authors declare that they have no competing interests.

## Authors’ contributions

I organized and prepare the framework of the study as a public health expert and Healthy City Coordinator of City of Aydin and wrote all manuscript. My assistants, OO and GS helped coordination of the study, entered the data and conducted the analyses. Assistant Professor FE and Professor EB gave contribution to the writing process and editorial assistance of this manuscript. All authors read and approved the final manuscript.

## Pre-publication history

The pre-publication history for this paper can be accessed here:

http://www.biomedcentral.com/1471-2458/12/437/prepub
